# Bile acid-containing lipid nanoparticles enhance extrahepatic mRNA delivery

**DOI:** 10.7150/thno.89913

**Published:** 2024-01-01

**Authors:** Savan K. Patel, Margaret M. Billingsley, Alvin J. Mukalel, Ajay S. Thatte, Alex G. Hamilton, Ningqiang Gong, Rakan El-Mayta, Hannah C. Safford, Maria Merolle, Michael J. Mitchell

**Affiliations:** 1 Department of Bioengineering, University of Pennsylvania, Philadelphia, PA 19104, USA.; 2 Perelman School of Medicine, University of Pennsylvania, Philadelphia, PA 19104, USA.; 3 Abramson Cancer Center, Perelman School of Medicine, University of Pennsylvania, Philadelphia, PA 19104, USA.; 4 Institute for Immunology, Perelman School of Medicine, University of Pennsylvania, Philadelphia, PA 19104, USA.; 5 Cardiovascular Institute, Perelman School of Medicine, University of Pennsylvania, Philadelphia, PA 19104, USA.; 6 Institute for Regenerative Medicine, Perelman School of Medicine, University of Pennsylvania, Philadelphia, PA 19104, USA.; 7 Penn Institute for RNA Innovation, Perelman School of Medicine, University of Pennsylvania, Philadelphia, PA 19104, USA.

**Keywords:** lipid nanoparticles, extrahepatic delivery, mRNA

## Abstract

Lipid nanoparticles (LNPs) have emerged as a viable, clinically-validated platform for the delivery of mRNA therapeutics. LNPs have been utilized as mRNA delivery systems for applications including vaccines, gene therapy, and cancer immunotherapy. However, LNPs, which are typically composed of ionizable lipids, cholesterol, helper lipids, and lipid-anchored polyethylene glycol, often traffic to the liver which limits the therapeutic potential of the platform. Several approaches have been proposed to resolve this tropism such as post-synthesis surface modification or the addition of synthetic cationic lipids.

**Methods:** Here, we present a strategy for achieving extrahepatic delivery of mRNA involving the incorporation of bile acids, a naturally-occurring class of cholesterol analogs, during LNP synthesis. We synthesized a series of bile acid-containing C14-4 LNPs by replacing cholesterol with bile acids (cholic acid, chenodeoxycholic acid, deoxycholic acid, or lithocholic acid) at various ratios.

**Results:** Bile acid-containing LNPs (BA-LNPs) were able to reduce delivery to liver cells *in vitro* and improve delivery in a variety of other cell types, including T cells, B cells, and epithelial cells. Our subsequent *in vivo* screening of selected LNP candidates injected intraperitoneally or intravenously identified a highly spleen tropic BA-LNP: CA-100, a four-component LNP containing cholic acid and no cholesterol. These screens also identified BA-LNP candidates demonstrating promise for other mRNA therapeutic applications such as for gastrointestinal or immune cell delivery. We further found that the substitution of cholic acid for cholesterol in an LNP formulation utilizing a different ionizable lipid, C12-200, also shifted mRNA delivery from the liver to the spleen, suggesting that this cholic acid replacement strategy may be generalizable.

**Conclusion:** These results demonstrate the potential of a four-component BA-LNP formulation, CA-100, for extrahepatic mRNA delivery that could potentially be utilized for a range of therapeutic and vaccine applications.

## Introduction

Messenger RNA (mRNA) therapies have emerged in the past decade as a promising strategy for a variety of applications ranging from protein replacement therapy to vaccination 1. The COVID-19 vaccines from Moderna and Pfizer/BioNTech were the first two FDA-approved mRNA drug products, and with their notable success, these vaccines demonstrate the immense potential of mRNA technologies 2. Though mRNA serves as a versatile clinical intervention, it is easily degraded in biological environments and thus, requires a delivery platform to reach the cytoplasm of target cells and achieve the functional outputs necessary for clinical efficacy 3. Both FDA-approved mRNA vaccines utilize ionizable lipid nanoparticles (LNPs) - composed of ionizable lipids, cholesterol, helper lipids, and lipid-anchored polyethylene glycol (PEG) - to encapsulate and deliver therapeutic mRNA via intramuscular (IM) injection 4,5. Beyond these vaccine technologies, LNP-based mRNA therapies are being explored for gene addition/replacement, gene expression control, and gene editing applications in a wide variety of diseases including congenital disorders, cancer immunotherapy, and corneal diseases 2,6-13.

For many of these applications, intravenous (IV) or intraperitoneal (IP) injections are preferred over IM to achieve systemic therapeutic impact or organ-specific delivery. However, there are drug delivery barriers that arise when attempting to deliver mRNA-containing LNPs (mRNA-LNPs) via IV or IP administration. Notably, IV-injected mRNA-LNPs experience significant accumulation and mRNA delivery to the liver. Two primary mechanisms have been proposed to explain this liver tropism: (1) LNPs bind to plasma proteins such as apolipoproteins that engage with hepatocyte cell receptors, enabling receptor-mediated uptake of LNPs and (2) the size of LNPs (~100 nm) subjects them to the hepatic first pass effect wherein drugs and drug carriers are trafficked through the liver vasculature 14-17. Furthermore, IP-injected mRNA-LNPs and liposomes experience similar trafficking to the liver as they undergo systemic absorption, indicating that the fate of IP-injected LNPs remains impacted by liver vasculature 18,19. Though hepatic delivery and expression of therapeutic mRNA are desired in certain applications (e.g., replacement plasma protein expression, gene editing for liver diseases, etc.), sufficient delivery to other organs (e.g., spleen, lungs, gastrointestinal tract, uterus, etc.) is necessary for the treatment of many diseases 20-25. Thus, these applications require an LNP platform that can avoid sequestration by the liver to achieve extrahepatic therapeutic efficacy.

Several methods have been developed to achieve extrahepatic mRNA-LNP delivery. First, ionizable lipid structure has been exploited as a strategy for organ-specific mRNA delivery and expression. Lipids such as C15 epoxide-modified low-molecular-weight polyethyleneimine (7C1) can achieve high amounts of lung endothelial mRNA expression 26,27. Furthermore, some researchers have developed degradable ionizable lipid structures that achieve splenic mRNA transfection 28. However, ionizable lipids are often proprietary, involve resource-intensive synthesis, typically require extensive development prior to incorporation into drug delivery systems, and have unclear mechanisms for achieving organ specificity 29-31. Alternatively, permanently charged lipids (SORT lipids) may be incorporated into LNP formulations in order to achieve differential mRNA expression in the spleen (using anionic lipids) or lungs (using cationic lipids) 30. Though potent in their organ tropism effects, these lipids must be added to existing formulation components, require chemical synthesis, may be toxic, and are expensive to acquire commercially 32. Other groups have proposed replacement helper lipids that are hypothesized to leverage similar charge-based mechanisms as the SORT lipids 33. However, these lipids are, once again, synthetic compounds, leading to the same potential limitations as SORT lipids. Finally, some groups have modified the surface of LNPs with targeting moieties (e.g., antibodies, antibody fragments, sugars, etc.) in order to drive receptor-mediated uptake in target cells and tissues 34,35. These ligand-based modification strategies require separate LNP synthesis and surface-modification steps, may require additional purification, add complexity to the regulatory profile of the LNP drug product, and can be costly, especially in the case of antibody-conjugated LNPs. Furthermore, surface-modified LNPs typically are not designed to avoid liver delivery, but rather to improve delivery to other organs or cell types. Moreover, some ligand modification strategies can prove to be immunogenic, complicating the translatability of surface-modified LNPs as a drug product 36. Therefore, there exists a need for a cost-effective and simple mRNA-LNP modification strategy to achieve extrahepatic delivery for IV and IP injection applications.

In this work, we explore a new class of cholesterol analogs - bile acids - in lipid nanoparticle formulations. Specifically, we investigate the effects of bile acid incorporation into LNPs on the relative expression of mRNA cargo in various organs following IV or IP injection. Previous work has investigated cholesterol analogs *in vitro* and *ex vivo* for their ability to hinder endosomal recycling within cells and improve transfection of mRNA cargo 37,38. For example, the use of hydroxycholesterols has been shown to improve delivery of luciferase-encoding mRNA to immortalized T cells and human primary T cells 39. This work explores bile acids - the final product of cholesterol metabolism that engages in an active exchange between the liver and gastrointestinal tract 40. Primary bile acids, such as cholic acid (CA) and chenodeoxycholic acid (CDCA), are produced by the liver and converted to secondary bile acids, such as deoxycholic acid (DCA) and lithocholic acid (LCA), by bacteria in the intestines 41. Therefore, following mRNA delivery, the bile acids within the BA-LNPs can be readily processed by the liver. Notably, bile acids are naturally present in blood at micromolar concentrations and can be commercially purchased in bulk quantities at lower costs than most synthetic lipids and cholesterol analogs, making them an appealing option for LNP formulation modification.

Therefore, we explored the effects of bile acids on functional mRNA biodistribution, using a library of 4 bile acid substitutes replacing cholesterol at varied replacement percentages. We screened these modified BA-LNP formulations *in vitro* and *in vivo* and identified CA-100 - a 4-component BA-LNP formulation, containing an ionizable lipid, helper lipid, PEG, and CA - that induced differential mRNA expression in the spleen following IV or IP injection. The replacement of cholesterol with CA, therefore, serves as a novel and promising LNP modification strategy to achieve functional extrahepatic mRNA delivery.

## Methods

### Lipid nanoparticle formulation

**Figure 1:**
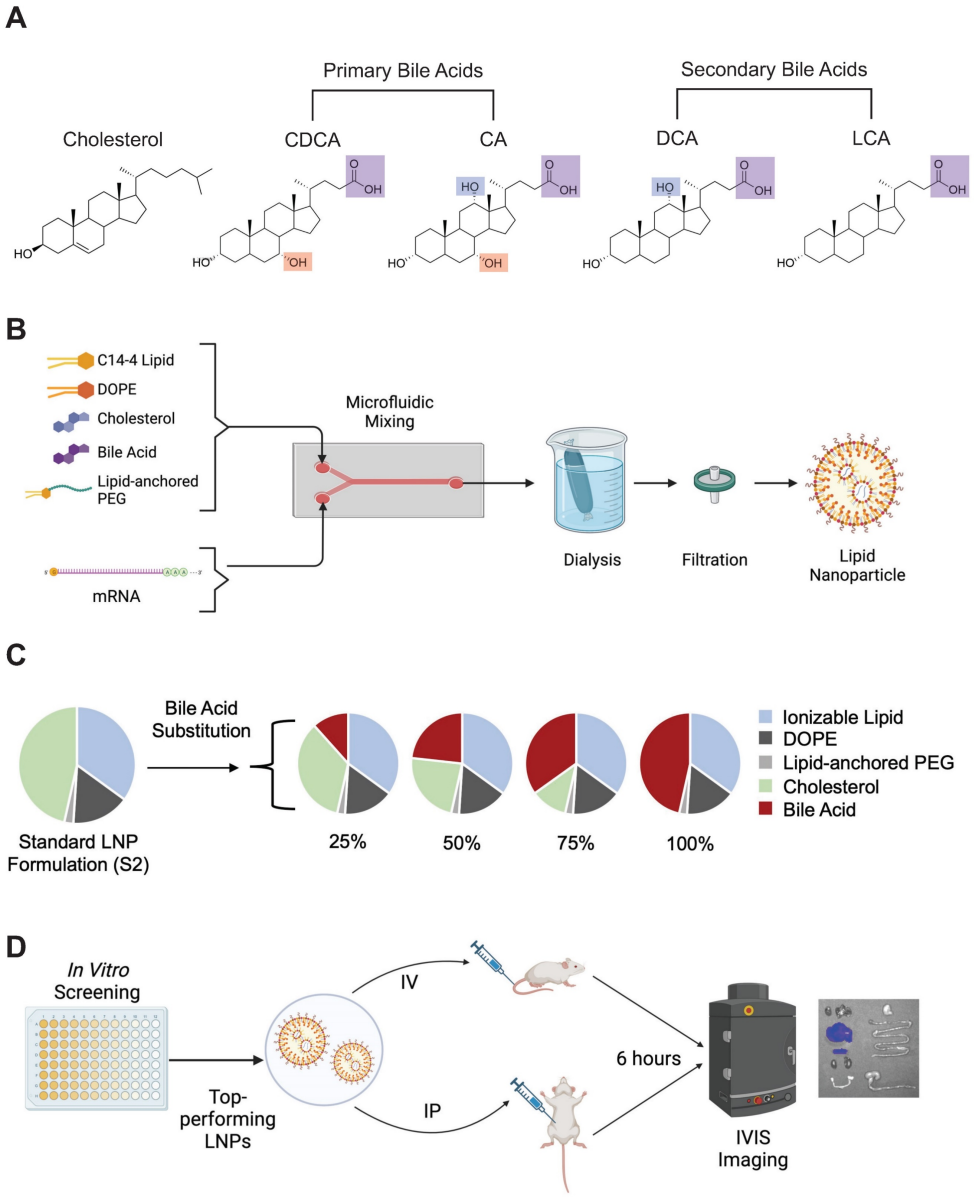
Bile acid-containing lipid nanoparticle (BA-LNP) design, synthesis, and optimization. (A) Structures of cholesterol, primary bile acids (chenodeoxycholic acid [CDCA] and cholic acid [CA]), and secondary bile acids (deoxycholic acid [DCA] and lithocholic acid [LCA]) with carboxylic acid groups highlighted in purple, C7 hydroxy groups highlighted in red, and C12 hydroxy groups highlighted in blue. (B) Schematic of LNP components, formulation, post-synthesis processing, and expected structure. (C) Design of an LNP library incorporating the substitution of various bile acids for unmodified cholesterol. (D) High throughput screening of LNPs *in vitro* to identify LNP formulation candidates for *in vivo* evaluation. Top-performing LNPs from the *in vitro* screen are assessed for biodistribution following either intraperitoneal (IP) injection or intravenous (IV) injection.

For ionizable lipids, either C14-4 or C12-200 (MedChemExpress, Monmouth Junction, NJ) were utilized in each formulation. C14-4 was synthesized as described previously 39. The remaining lipid components were composed of various ratios of 1,2-Dioleoyl-sn-glycero-3-phosphoethanolamine (DOPE) (Avanti Polar Lipids, Alabaster, AL), 1,2-dimyristoyl-sn-glycero-3-phosphoethanolamine-N-[methoxy(polyethylene glycol)-2000] (PEG) (Avanti Polar Lipids), cholesterol (Avanti Polar Lipids), and bile acids. The bile acids used in this study were cholic acid (CA) (Sigma Aldrich, St. Louis, MO), chenodeoxycholic acid (CDCA) (Sigma Aldrich), deoxycholic acid (DCA) (Sigma Aldrich), and lithocholic acid (LCA) (Sigma Aldrich) (**Figure 1A**). All lipid components were suspended in ethanol.

CleanCap® FLuc mRNA (5moU) (TriLink Biotechnologies, San Diego, CA) encoding luciferase protein was diluted in 10 mM citric acid at 25 µg mRNA to 300 µL solvent. Using pump33DS syringe pumps (Harvard Apparatus, Holliston, MA), the ethanol (lipid) phase and aqueous citric acid (mRNA) phases were chaotically mixed in a microfluidic device at a 1:3 volume ratio to formulate LNPs 42. Then, LNPs were dialyzed using 20 kDa molecular weight cutoff dialysis cassettes for 2 hr against 1X PBS. Finally, LNPs were filtered using 0.22-micron filters (**Figure 1B**).

### LNP library design

A base formulation (S2) containing, by molar ratio, 35% ionizable lipid (either C14-4 or C12-200), 16% DOPE, 46.5% cholesterol, and 2.5% PEG was used as a control for all experiments. A library was generated from this base formulation wherein the excipient molar ratios were maintained. Cholesterol was substituted at various molar percentages (25%, 50%, 75%, and 100%) by different bile acids (CA, CDCA, DCA, or LCA) such that the combined amount of cholesterol and bile acid in any given formulation summed to 46.5% of the total lipid molar quantity (**Figure 1C**). Each candidate in the library was named by the bile acid substitute and the percentage substitution. For example, a formulation containing 25% cholesterol and 75% CDCA (i.e., 75% substitution), was named CDCA-75.

### LNP characterization

LNP z-average diameter (size) and polydispersity index (PDI) were determined using dynamic light scattering with the Zetasizer Nano (Malvern Instruments, Malvern, UK). Samples were prepared in a cuvette following a 1:100 dilution in 1X PBS. LNP zeta potential was measured using the Zetasizer Nano. Samples were prepared in DTA1070 zeta potential cuvettes (Malvern Panalytical, Malvern, UK) following a 1:50 dilution in water. For all *in vitro* and *in vivo* dosing, LNP mRNA concentration was determined via A260 absorbance on an Infinite M Plex plate reader (Tecan, Morissville, NC).

LNP mRNA encapsulation efficiency was determined with the Quant-iT™ RiboGreen™ RNA Assay Kit (Thermo Fisher Scientific, Waltham, MA). LNP samples were diluted to 2 µg/mL in 1x TE buffer or 0.1% Triton X-100 in 1x TE buffer (Sigma Aldrich). Following incubation for 20 minutes at 300 rpm, 100 µL of each solution and 100 µL of RiboGreen™ reagent were added to wells on a 96-well plate. Fluorescence readings from each well were recorded using the Infinite M Plex plate reader (Tecan, Morissville, NC) at an excitation of 490 nm and an emission of 520 nm. Encapsulation efficiency is reported as a calculated value: 1 - RT / RX where RT is the RNA content in the TE buffer and RX is the RNA content in Triton X-100 buffer.

pKa of the LNPs was determined using 6-(p-Toluidino)-2-naphthalenesulfonic Acid (TNS) assays. Solutions ranging from pH values of 2 to 12 in 0.5 increments buffered with 150 mM sodium chloride, 20 mM sodium phosphate, 25 mM ammonium citrate, and 20 mM ammonium acetate were prepared. In a 96-well plate, LNPs were incubated with the various pH solutions and 6 µM TNS for 20 minutes on a shaker. Finally, the fluorescence was read on the Infinite M Plex plate reader and the resulting data was fit with a sigmoidal curve. The best-fit curve was then used to approximate the pKa of the LNP sample by determining the pH at which the fluorescence values reached 50% of their maximum.

Cryo-electron microscope (cryo-EM) images were captured to characterize LNP morphology and structure. 3 μl of LNPs at approximately 150 ng/μl were applied to glow-discharged QUANTIFOIL® Holey Carbon grids. Grids were blotted and plunged into liquid ethane using a Vitrobot Mark IV (Thermo Fisher Scientific). Imaging was performed on a Titan Krios (FEI Company, Hillsboro, OR) equipped with a K3 Bioquantum at the Beckman Center (University of Pennsylvania, Philadelphia, PA).

### Cell culture, mRNA-LNP treatment, viability assays, and expression assays

HeLa (ATCC no. CCL-2) cells, HepG2 (ATCC no. HB-8065), and Caco-2 (ATCC no. HTB-37) were cultured in Dulbecco's Modified Eagle Medium (Thermo Fisher Scientific) supplemented with 10% fetal bovine serum (FBS) and 1% penicillin-streptomycin (P/S). Jurkats (ATCC no. TIB-152) and Raji (ATCC no. CCL-86) were cultured in RPMI-1640 with L-glutamine (Thermo Fisher Scientific) supplemented with 10% FBS and 1% P/S. For *in vitro* screening, the various cell lines were plated in 96-well plates and treated with mRNA-LNPs at previously optimized cell densities and mRNA concentrations. HeLa, HepG2, Caco-2, Jurkats, and Raji cells were plated at 10, 5, 25, 60, and 60 thousand cells per well, respectively. Furthermore, they were treated with 10, 10, 100, 60, and 60 ng of mRNA, respectively. The mRNA concentration of LNPs was determined using A260 absorbance with Tecan's NanoQuant plate on an Infinite M Plex plate reader. After 24 hours of incubation, cells were read out using functional readout assays.

For luciferase expression assays involving non-adherent cell lines (Jurkats and Raji), 96-well plates were first centrifuged at 300 g for 5 min to pellet cells. For all cell lines, supernatant media was removed, and cells were resuspended in 100 μL of luciferase assay substrate (Promega) and 50 μL 1× lysis buffer (Promega, Madison, WI). After 10 minutes, luminescent signal from each well was read on the Infinite M Plex plate reader (**Figure 1D**). Luminescence was normalized within each plate to the S2 LNP formulation.

For cell viability assays, 60 μL of CellTiter-Glo™ (Promega) was added to each well for all cell lines. After 10 minutes, luminescent signal from each well was read on the Infinite M Plex plate reader (**Figure 1D**). Luminescence was normalized within each plate to untreated cells to calculate percent cell viability.

### *In vivo* experiments and imaging

All animal protocols were approved by the Institutional Animal Care and Use Committee of the University of Pennsylvania (#806540), and all performed procedures were in accordance with the Guidelines for Care and Use of Laboratory Animals at the University of Pennsylvania. For all *in vivo* experiments, C57BL/6 female mice (6-8 weeks of age, ~20 g body weight) were utilized. mRNA-LNP solutions were concentrated prior to use in animal experiments using Amicon Ultra-15 Centrifugal Filter Unit (Sigma Aldrich) with 50 kDa filters until they reached ~100 ng mRNA / μL. Mice were then injected at 1 mg mRNA / kg body weight (~200 μL) of the concentrated mRNA-LNP solutions either intraperitoneally (IP) or intravenously (IV) (**Figure 1D**). After 6 hours, mice were injected IP with 200 μL of luciferin reagent (15 mg/mL). Following 10 minutes of incubation, the mice were euthanized with CO_2_ and organs (heart, lungs, liver, spleen, kidneys, uterus, stomach, small intestine, and large intestine) were dissected and imaged for bioluminescence using an IVIS imaging system (PerkinElmer, Waltham, MA) (**Figure 1D**). Total flux is reported after background signal from each image was subtracted. Background signal is defined as the average flux measured on PBS-treated mice.

In the Cre/lox model, Ai9 female mice were utilized. mRNA-LNP solutions were prepared and concentrated as described earlier, but instead using Cre recombinase-encoding CleanCap® Cre mRNA (5moU) (TriLink Biotechnologies). Mice were injected at 0.5 mg mRNA / kg body weight (~200 μL) of concentrated mRNA-LNP solution. After 3 days, the mice were euthanized with CO_2_ and spleens were dissected.

For accumulation studies, mRNA-LNPs were incubated with 1% DiR (Thermo Fisher Scientific) by volume for 30 minutes. They were subsequently concentrated using 50 kDa filter conical tubes as previously described. In addition to bioluminescent readings, fluorescence readings were obtained using the IVIS imaging system (PerkinElmer).

### Spleen processing and flow cytometry

Spleens were homogenized through cell strainers to form a single-cell suspension in 0.5% PBSA. Red blood cells were lysed out of samples with red blood cell lysis buffer (Invitrogen, Waltham, MA).

Single-cell suspensions of the spleen were then incubated with TruStain FcX™ (Biolegend, San Diego, CA) according to manufacturer instructions. Samples were then stained with 1.5 tests each of various antibodies according to manufacturer instructions: Brilliant Violet 421™ anti-mouse F4/80 Antibody (Biolegend), Alexa Fluor® 488 anti-mouse CD19 Antibody (Biolegend), Brilliant Violet 605™ anti-mouse CD11c Antibody (Biolegend), APC anti-mouse CD3 Antibody. All samples were washed twice with cold PBSA before being run on a BD LSR II Flow Cytometer (BD Biosciences, Macquarie Park, NSW, Australia). Standard gating was performed for doublet exclusion and cell populations were identified as positive for their marker and negative for two other markers in the stain. Since dendritic cells were lowest in abundance, they were not used to negatively gate any of the other cell subsets. T cells were gated as CD19-/F480-/CD3+. B cells were gated as CD3-/F480-/CD19+. Macrophages were gated as CD3-/CD19-/F480+. Dendritic cells were gated as CD3-/CD19-/CD11c+. Finally, tdTomato fluorescence was used to determine functional mRNA delivery. Spectral overlap compensation, gating, and analysis were performed using FlowJo™ (BD Biosciences).

## Results

### LNP library design, formulation, and characterization

The goal of this study was to explore the effects of cholesterol analogs, namely bile acids, on LNP function. Here, 4 bile acid candidates were evaluated for their impact on LNP physiochemical properties and mRNA delivery: CA, CDCA, DCA, and LCA. There are several chemical characteristics of these bile acid candidates that may impact the resulting LNP. First, compared to unmodified cholesterol, all four bile acids have a carboxylic acid group at the tail of the sterol molecule which enables them to act as acids in aqueous environments. This could potentially alter the pKa of the resulting LNP, thereby affecting the endosomal processing of the LNPs. Second, all four bile acids lack the double bond between C5 and C6 which can influence sterol-lipid interactions in membranes 43. Finally, the addition of hydroxy groups on various carbon atoms in the sterol has also been shown to influence LNP membrane stability and mRNA delivery 39. These hydroxy groups add hydrophobicity to the respective domains of the sterol molecule, thereby impacting the modified sterol's ability to align itself in lipid membranes. The primary bile acids, CA and CDCA, have an additional hydroxy group located on C7, highlighted in red (**Figure 1A**). Interestingly, our previous work demonstrated that LNPs containing 7α-hydroxycholesterol, a modified cholesterol with a hydroxy group on C7, improved mRNA delivery to T cells *in vitro* and *ex vivo* and altered endosomal processing 39. CA and DCA also have an additional hydroxy group on the C12 carbon, highlighted in blue (**Figure 1A**).

The various modifications that constitute these bile acids made them interesting candidates to evaluate for their impact on LNP physiochemical properties and subsequent mRNA delivery. Therefore, a library of 16 LNPs and 1 control LNP, S2, was synthesized (**Figure 1B**). The 16-LNP library is composed of LNPs with various substitution percentages (25%, 50%, 75%, and 100%) of the 4 bile acids (**Figure 1C**). In each formulation, unmodified cholesterol was replaced by the selected bile acid at the given substitution percentage. LNPs were produced using chaotic mixing in microfluidic devices and underwent dialysis with phosphate-buffered saline (PBS) after formulation before being used for cell treatment, animal experiments, or characterization (**Figure 1D**). Importantly, all bile acids were incorporated into the lipid mixtures and underwent chaotic mixing with all other lipid components (ionizable lipids, cholesterol, DOPE, and lipid-anchored PEG).

Given that bile acids were incorporated directly into the LNP formulation process, we hypothesized that their presence and the simultaneous reduction of unmodified cholesterol during LNP formulation may influence LNP formation and the resulting physiochemical properties of the LNPs. This was even more likely given the chemical differences between cholesterol and each of the bile acids. Specifically, the amphiphilic nature of cholesterol enables it to aid in the alignment of other lipids within the LNP membrane 44. The various functional groups on the bile acid candidates alter the hydrophobicity profile of each molecule relative to unmodified cholesterol. Therefore, to assess these effects, we evaluated LNP size, polydispersity (PDI), mRNA encapsulation efficiency, zeta potential, and pKa to characterize the LNP library (**Table 1**).

**Figure 2:**
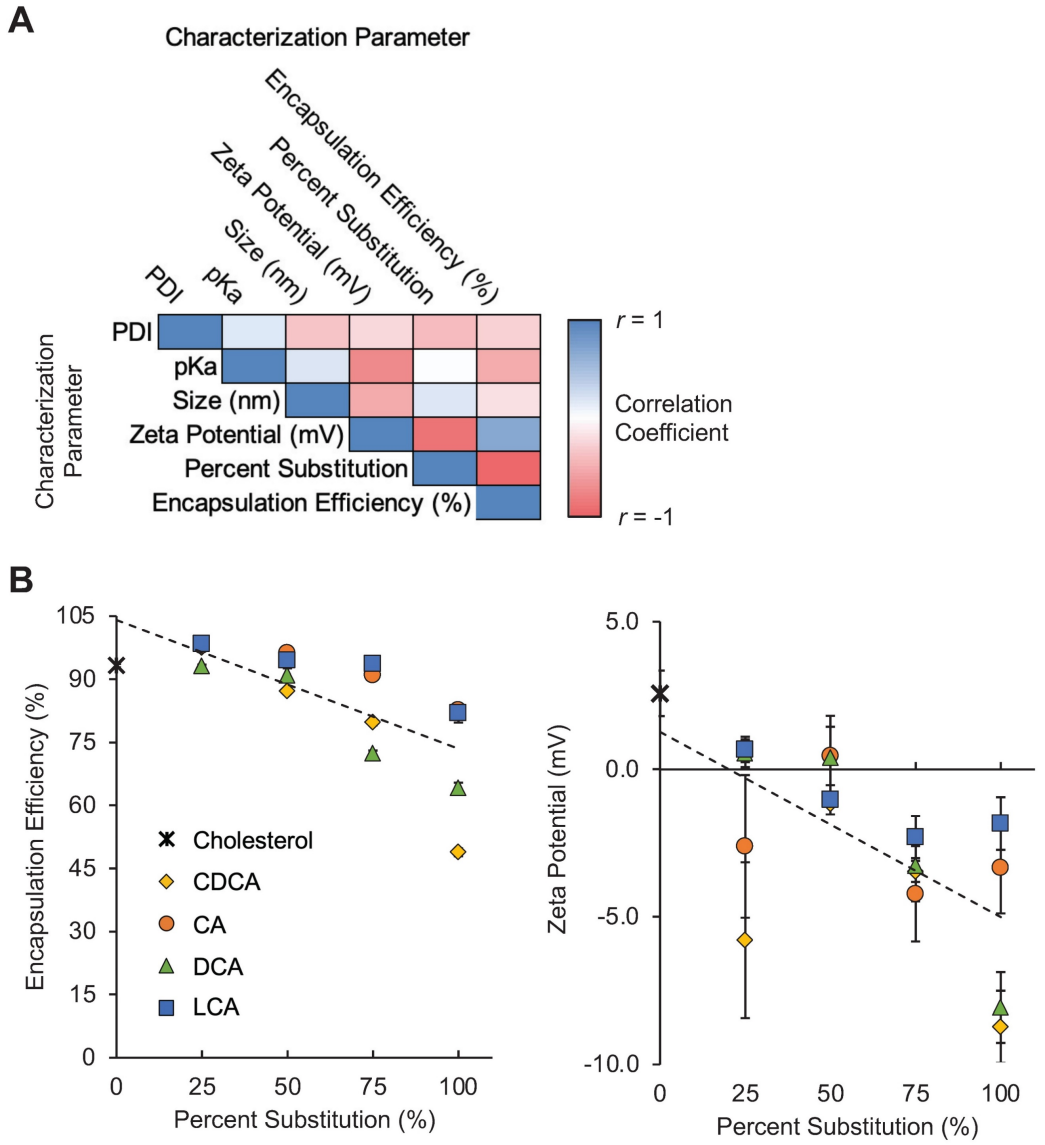
Characterization of LNP formulations containing various amounts of bile acids. (A) Correlation matrix of characterization parameters for the 17 LNPs in the library (16 BA-LNP formulations and 1 base formulation). LNPs were grouped by percentage substitution (0, 25, 50, 75, or 100%). Measured characterization parameters include hydrodynamic size, PDI, encapsulation efficiency, zeta potential, and pKa. n = 3 for all measured characterization parameters. (B) Scatter plots of encapsulation efficiency (left) and zeta potential (right) versus bile acid substitution percentage for the LNP library. Least squares linear regression lines were used to visualize trends. n = 3. Error bars denote standard deviation.

When grouped by percentage substitution, the LNPs in our library appear to demonstrate moderate negative correlations between percent substitution and mRNA encapsulation efficiency and between percent substitution and zeta potential (**Figure 2A**). These findings suggest that the substitution of bile acids, in general, for cholesterol in LNP formulations may reduce the ability of LNP formulations to encapsulate mRNA, possibly by altering lipid membrane formation (**Figure 2B**). Furthermore, the addition of bile acids, which contain carboxylic acid groups that become negatively charged following deprotonation, appears to reduce the zeta potential of LNPs (**Figure 2B**). Other groups have shown that the addition of cationic or anionic lipids to LNP formulations alters LNP zeta potential and drives differential LNP biodistribution 30. We hypothesized that this shift in LNP charge as a result of bile acid incorporation may result in altered LNP biodistribution in animal models via a similar mechanism of action.

### *In vitro* LNP library screening

To evaluate if the incorporation of bile acids into LNP formulations results in differences in cell-specific uptake, we screened this library in various cell lines for their ability to deliver luciferase-encoding mRNA. Luciferase-encoding mRNA is translated in cells following cytosolic delivery, and the resulting luciferase protein reacts with luciferin reagent to produce a luminescent output that corresponds to mRNA delivery. HeLa cells were utilized as a standard cell line, Caco-2 intestinal epithelial cells were used to represent epithelial tissue, and HepG2 cells were used to represent the liver. Additionally, Jurkat and Raji cells were used to predict T and B cell (i.e., lymphocyte) delivery, respectively. These cell lines were selected for their ability to model potential *in vivo* therapeutic targets or organs of interest. HeLa cells were selected to model general, non-specialized cell uptake. Caco-2 cells were used as the intestinal epithelium is responsible for several gastrointestinal disorders and is lined with vasculature which may enable mRNA-LNPs to reach this tissue for the treatment of diseases of the gut and colon. HepG2 cells were used to model hepatocytes, which constitute ~80% of the liver, as this immortalized cell line retains metabolic activity like that of hepatocytes 45. HepG2 cells are also extensively utilized in the literature for evaluating drug/nanoparticle metabolism and hepatotoxicity 46. Finally, the immortalized T and B cell lines were utilized as lymphocytes are a target for immunotherapies. In this screen, each of these cell lines was treated with formulations from the LNP library and the resulting luminescent signal was measured as compared to cells treated with S2.

**Figure 3:**
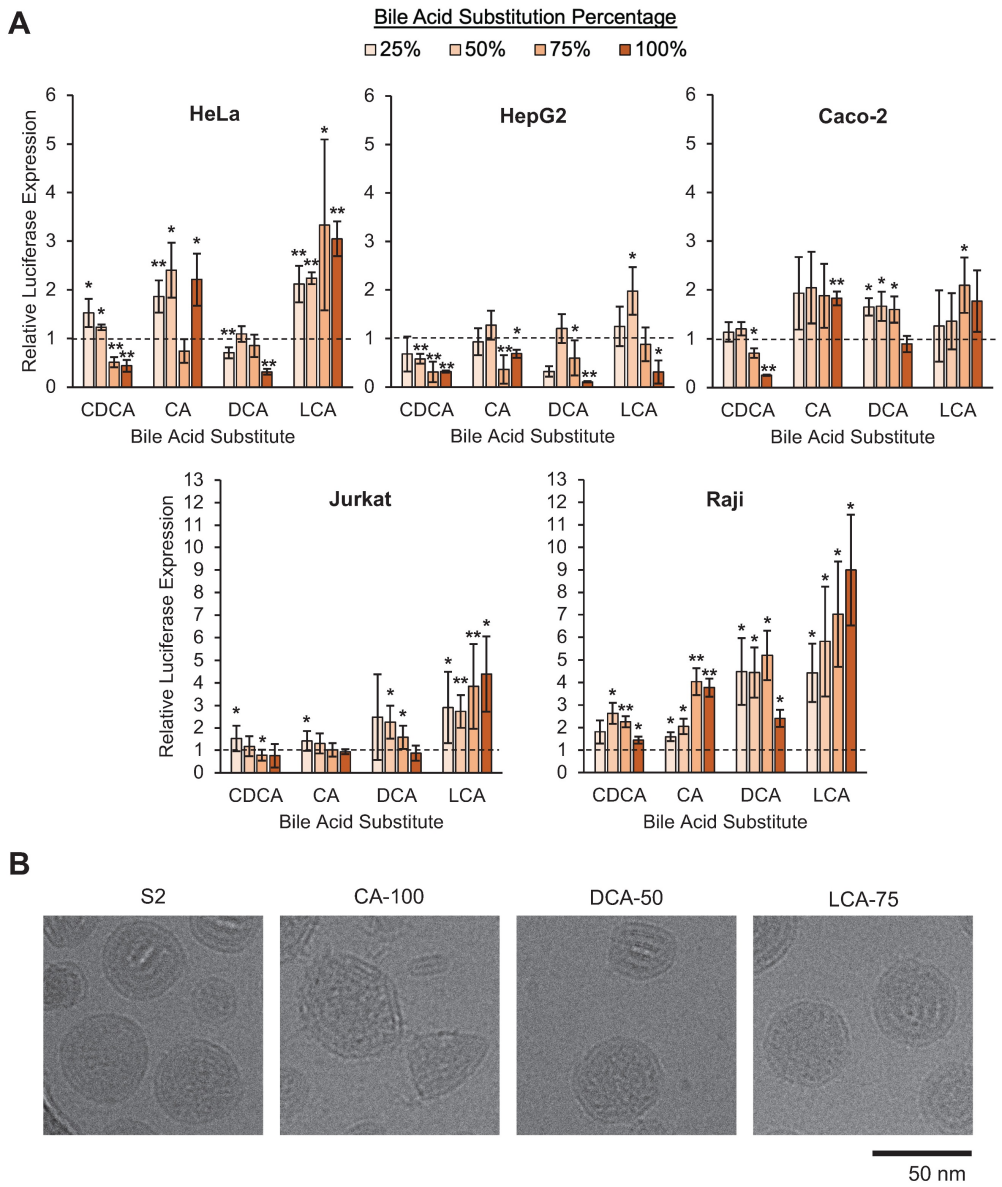
*In vitro* screening of the LNP library and morphologic characterization of selected candidates. (A) Luciferase mRNA delivery in various cell lines. Luciferase expression was normalized to cells treated with S2, the base formulation, after background was subtracted. HeLa cervical cancer cells were treated at 10 ng mRNA / 10,000 cells. HepG2 hepatocytes were treated at 10 ng mRNA / 5,000 cells. Caco-2 intestinal epithelial cells were treated at 100 ng mRNA / 25,000 cells. Jurkat T cells and Raji B cells were treated at 60 ng mRNA / 60,000 cells. Legend denotes percent substitution of each bile acid into the S2 formulation. n = 3 biological replicates. Error bars denote standard deviation. An ANOVA was used to determine if treatment group means differed significantly. *: *p*<0.05. **: *p*<0.01 in a *post hoc* Student's t-test between LNP candidate and S2. (B) Representative cryo-electron microscopy images of S2, CA-100, DCA-50, and LCA-75 to identify morphological variation amongst selected LNPs.

When comparing the LNP library's mRNA delivery across cell lines, there were notable trends in the impact of bile acid substitutions on mRNA delivery. In HeLa and Caco-2 cells, increases in delivery were modest, as some formulations increased luminescence signal between 1.5-fold and 3-fold. In HepG2 cells, luminescence was reduced in most bile acid-containing LNPs (BA-LNPs) relative to the control, S2 (**Figure 3A**). Interestingly, in the two lymphocyte cell lines, Jurkat (T cells) and Raji (B cells), several BA-LNP formulations, particularly those containing DCA and LCA (secondary bile acids), were able to achieve luminescent readouts demonstrating 6-fold to 10-fold improvements, nearly an order of magnitude improvement (**Figure 3A**). Despite these significant improvements in functional mRNA delivery, BA-LNPs did not induce cytotoxicity beyond that of the unmodified LNP (S2) (**Figure S1**).

Though *in vitro* results often do not extrapolate to *in vivo* organ-level and tissue-level delivery, we utilized these screen results to determine a subset of promising bile acid-containing LNPs to move forward with for *in vivo* experiments. Since we were interested in achieving differential delivery to extrahepatic organs, we identified and selected LNP formulations that (1) improved delivery in non-liver cells and (2) reduced/maintained delivery in the liver cell line as a potential predictor of reduced *in vivo* liver delivery.

During this selection of BA-LNP candidates, no CDCA-containing candidates were selected as they did not improve mRNA delivery across the various cell lines. In lymphocyte populations, CDCA-containing LNPs had the lowest improvements in mRNA delivery. Although CDCA-25 and CDCA-50 had slight improvements in HeLa mRNA delivery, Caco-2 mRNA delivery was not improved. Amongst CA-containing LNPs, CA-100 was chosen over other candidates because it had substantial Caco-2 mRNA delivery improvements, potentially indicating increased mRNA delivery to epithelial and epithelial-like tissue. Furthermore, we were interested in including a bile acid-containing LNP that had a 100% substitution of bile acid, in this case, CA, to explore how LNPs lacking cholesterol performed in biological environments. From the DCA-containing LNPs, DCA-50 was chosen as a potential negative control given that it had close to baseline levels of luminescence in HepG2 cells and moderate delivery to other cell lines, including lymphocyte populations. Finally, from LCA-containing LNPs, LCA-75 was chosen as it had the highest HeLa mRNA delivery, representing general cell delivery. Furthermore, it had significantly improved Caco-2 mRNA delivery and substantial lymphocyte mRNA delivery with no significant change to HepG2 delivery potentially indicating differential uptake in non-liver cells. Therefore, based on this reasoning, all future screens were focused on 3 bile acid-containing LNPs (CA-100, DCA-50, and LCA-75) along with our control LNP containing only unmodified cholesterol (S2).

### Cryo-EM of selected LNP candidates

The selected BA-LNPs, CA-100, DCA-50, and LCA-75, along with the control LNP, S2, were imaged using cryo-electron microscopy to determine if the incorporation of bile acids induced morphological changes to the resulting LNPs given cholesterol's role in lipid membrane alignment. Notably, as compared to the mostly spherical S2 control LNPs, the CA-100 formulation exhibits a polymorphism with several sharp corners (**Figure 3B**). The representative image also captures a small capsule-shaped aggregate that does not appear to contain mRNA which, in conjunction with the reduced encapsulation efficiency observed in our characterization studies, may indicate a reduced ability for CA-containing LNPs to encapsulate mRNA. In DCA-50 and LCA-75, the presence of bile acid with some amount of cholesterol still appears to result in the formation of corners and polygonal structure, although to a lesser degree than CA-100 (**Figure 3B**). Previous studies have also found that LNPs that exhibit polymorphisms can alter the intracellular delivery of mRNA 37. Therefore, any differential delivery observed *in vitro* or *in vivo* may be partially explained by morphology-driven mechanisms.

### Biodistribution of BA-LNPs following intraperitoneal injection

The peritoneal cavity contains several organs of interest for gene delivery applications including the spleen, kidneys, gastrointestinal tract, uterus, and liver 47. In addition to providing exposure to the exterior of these organs, IP injection can also achieve systemic therapeutic effects more gradually than IV injection which may generate a favorable pharmacokinetic profile for certain therapeutic applications. Specifically, IP injection can avoid the initially high concentrations of drug or therapeutic that IV injection experiences by slowly allowing the therapeutic to enter the vasculature 48,49. As such, we were interested in evaluating the biodistribution of functional mRNA delivery following IP injection of our selected LNP candidates (S2, CA-100, DCA-50, and LCA-75) in mice.

**Figure 4:**
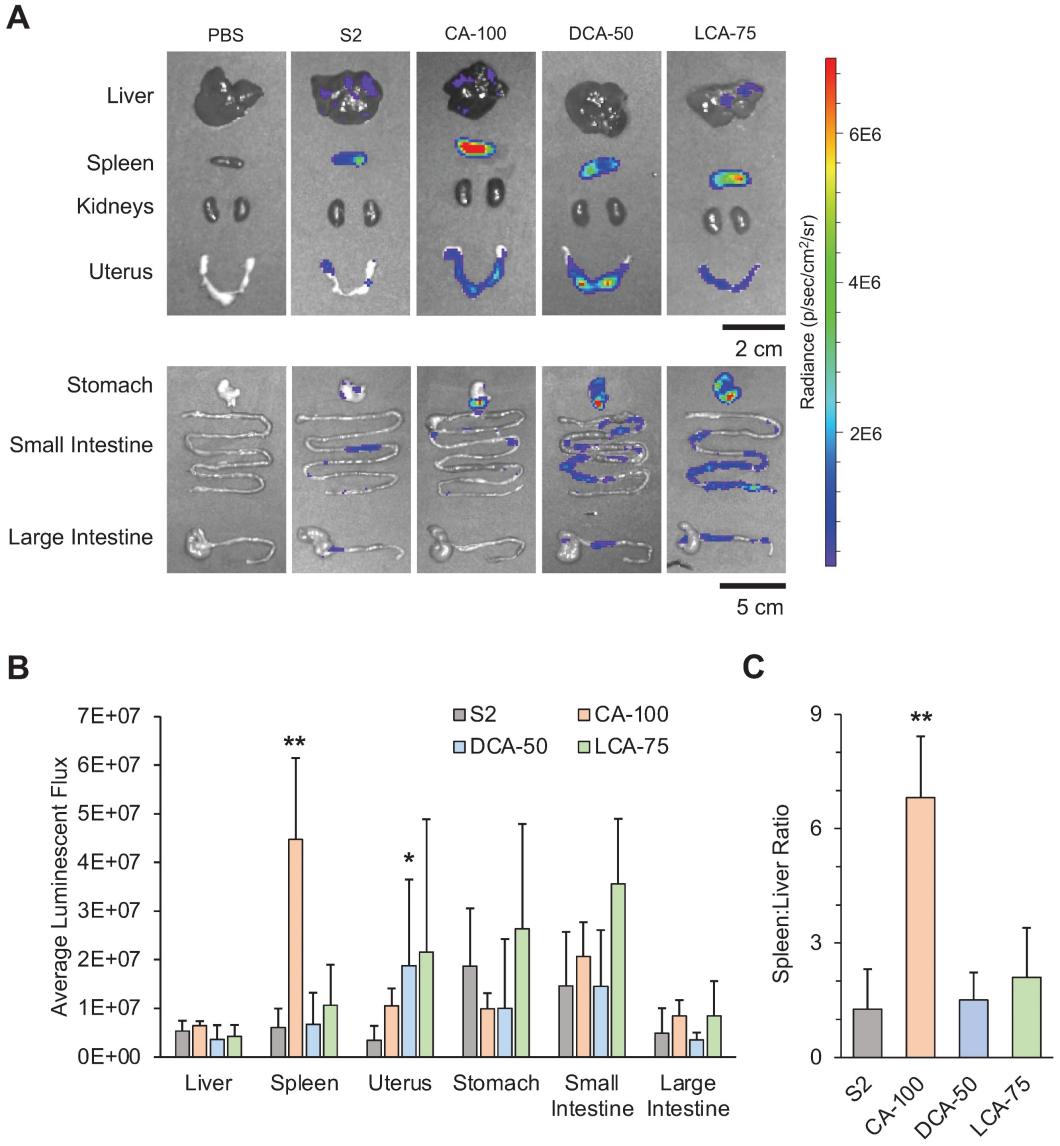
Luciferase mRNA delivery following intraperitoneal injection (1 mg mRNA / kg) of LNPs into mice. Mice were dissected and imaged 6 hours after treatment. (A) Representative IVIS images of mouse organs from each treatment group (PBS, S2, CA-100, DCA-50, and LCA-75). (B) Quantification of total luminescent flux in several organs of the peritoneal cavity (liver, spleen, uterus, stomach, small intestine, and large intestine) following IP injection with S2, CA-100, DCA-50, or LCA-75. Total flux is reported after subtracting background signal from each image. An ANOVA was used to determine if treatment group means differed significantly. (C) Liver-to-spleen total luminescent flux ratios for S2, CA-100, DCA-50, and LCA-75 treatment groups. n = 3 mice per group. Error bars denote standard deviation. For all statistical tests, *: *p*<0.05. **: *p*<0.01 in a *post hoc* Student's t-test between LNP candidate and S2.

Following IP injection with the selected LNP candidates containing luciferase-encoding mRNA, organs of the peritoneal cavity were dissected after 6 hours to assess biodistribution (**Figure 4A**). Comparisons of luminescent expression were made for each organ between each of the different treatment groups and the control treatment (S2) to determine if any BA-LNPs exhibited differential delivery to any organ. For most organs, the BA-LNPs did not differ from the cholesterol-only LNP in delivery to the various organs. However, CA-100 had significant increases in mRNA delivery to the spleen and DCA-50 had significant increases in mRNA delivery to the uterus relative to S2 (**Figure 4B**). Notably, the magnitude of CA-100 splenic delivery relative to S2 was nearly 4-fold. Thus, as CA-100 did not change mRNA expression in the liver relative to S2, but successfully increased expression of mRNA in the spleen, its distribution has demonstrated enhanced extrahepatic tropism.

To evaluate the degree of CA-100 splenic tropism, we compared the ratio of spleen luminescence to liver luminescence. In some disease models, it is therapeutically advantageous to maximize delivery to the spleen, while minimizing off-target delivery to the liver 50. Thus, quantifying tropism to the spleen relative to the liver (i.e., spleen:liver ratio) can be useful for screening LNP formulations. A greater spleen:liver ratio would represent an LNP candidate that can achieve splenic mRNA delivery without simultaneously saturating the liver with mRNA. CA-100 achieved a spleen:liver ratio greater than 6 while S2 had a spleen:liver ratio near 1 (**Figure 4C**). This suggests that CA-100 may serve as an LNP candidate for splenic mRNA delivery via intraperitoneal injection. Importantly, CA-100 only contains CA, thus the replacement of cholesterol with CA in this formulation was the driving factor in the splenic tropism following IP injection.

In addition to the splenic tropism of CA-100, there appears to be delivery of luciferase-encoding mRNA to the small intestine and stomach with all BA-LNP candidates, particularly LCA-75. Further, LCA-75 achieved higher absolute luminescence in all evaluated extrahepatic organs and slightly reduced absolute luminescence in the liver relative to S2. However, statistical comparisons reveal that these improvements are not statistically significant at the current mRNA dosage. Therefore, future research may investigate if this trend may be exploited, perhaps at higher dosages, to achieve delivery to the gastrointestinal tract.

### Biodistribution of BA-LNPs following intravenous injection

Though IP injection of mRNA-LNPs is a desirable strategy for certain clinical applications, we wanted to explore the biodistribution of the BA-LNPs following IV administration to evaluate their ability to serve as delivery vehicles for mRNA therapeutics that require the rapid onset and predictable pharmacological profile of IV administration for maximum therapeutic effect 51. However, intravenous injection poses its own problems for mRNA and nanotherapeutics as blood maintains an enzymatic environment that also induces degradation. Furthermore, plasma proteins such as apolipoprotein E (ApoE) are known to bind to LNPs, form a protein corona, and drive receptor-mediated uptake in the liver which limits delivery to extrahepatic organs 52. Notably, cholesterol plays an important role in enabling the formation of a protein corona 53. Therefore, since our selected bile acid-containing LNPs lack or have reduced levels of cholesterol, we hypothesized that the protein corona and subsequent corona-mediated uptake may be altered.

**Figure 5:**
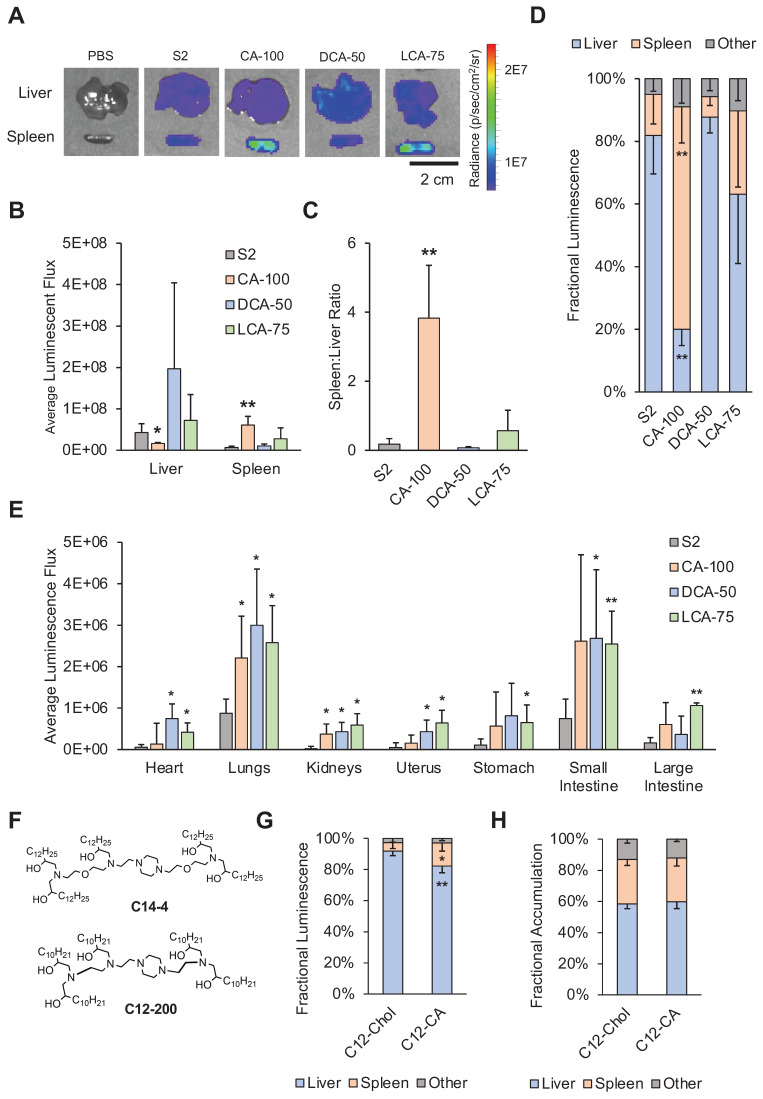
Biodistribution studies following intravenous injection of LNPs into mice. For (A) - (F), mice were injected with 1 mg luciferase mRNA / kg and then dissected and imaged 6 hours after treatment. (A) Representative IVIS images of liver and spleen from each treatment group (PBS, S2, CA-100, DCA-50, and LCA-75). (B) Quantification of total luminescent flux in the liver and spleen following IV injection with S2, CA-100, DCA-50, or LCA-75. (C) Liver-to-spleen total luminescent flux ratios for S2, CA-100, DCA-50, and LCA-75 treatment groups. (D) Fractional distribution of luminescence amongst organs (liver, spleen, and all other imaged organs) for mice treated with S2, CA-100, DCA-50, or LCA-75, where other imaged organs include heart, lungs, kidneys, uterus, stomach, small intestine, and large intestine. (E) Quantification of total luminescent flux in heart, lungs, kidneys, uterus, stomach, small intestine, and large intestine following IV injection with S2, CA-100, DCA-50, or LCA-75. (F) Chemical structures of ionizable lipids used in LNP formulations: C14-4 and C12-200. For (G) - (H), mice were injected with DiR-labeled C12-200 LNPs at 1 mg luciferase mRNA / kg and then dissected and imaged 6 hours after treatment. (G) Fractional distribution of luminescence amongst organs (liver, spleen, and all other imaged organs) for mice treated with C12-Chol or C12-CA. (H) Fractional distribution of LNP accumulation, measured by fluorescent signal of DiR, amongst organs (liver, spleen, and all other imaged organs) for mice treated with C12-Chol or C12-CA, where other imaged organs include heart, lungs, kidneys, uterus, stomach, small intestine, and large intestine. For all experiments, n = 4 mice per group. Error bars denote standard deviation. For all statistical tests, *: *p*<0.05. **: *p*<0.01 in a *post hoc* Student's t-test between LNP candidate and S2. Where applicable, an ANOVA was first used to determine if treatment group means differed significantly.

To assess the biodistribution of our selected bile acid-containing LNP candidates, we intravenously injected these LNPs and a control LNP (S2, CA-100, DCA-50, and LCA-75) encapsulating luciferase-encoding mRNA into mice. Organs of the peritoneal cavity in addition to the heart and lungs, were dissected after 6 hours and evaluated for luminescence using IVIS (**Figure 5A**). Though some increased mRNA delivery was observed in all the organs using BA-LNPs as compared to the S2 LNPs, luminescent expression in both the liver and spleen is nearly two orders of magnitude greater than all other harvested organs for most LNP candidates. Thus, most of the results explored further here focus on delivery in these two organs.

Though all tested LNPs led to delivery primarily to the spleen and liver, each LNP formulation displayed different distributional patterns in this delivery. CA-100, the same LNP that improved spleen delivery following IP injection, significantly reduced luminescence in the liver and increased luminescence in the spleen relative to S2 (**Figure 5B**). DCA-50 and LCA-75, which, unlike CA-100, both contain a fraction of cholesterol, did not significantly change spleen or liver delivery relative to S2. As in the IP data analysis, we wanted to further quantify the various tropisms observed with the BA-LNP candidates. S2, DCA-50, and LCA-75 had spleen:liver ratios below 1, implying that they primarily achieve liver delivery (**Figure 5C**). However, CA-100 achieved a spleen:liver ratio of approximately 4 which demonstrates greater spleen mRNA expression than liver mRNA expression, indicating primarily spleen delivery. Importantly, S2's mRNA delivery to the liver was 6-fold greater than its mRNA delivery to the spleen while CA-100's mRNA delivery to the spleen 4-fold greater than its mRNA delivery to the liver. This reversal of delivery profile due to the complete replacement of cholesterol with CA further highlights CA-100 spleen tropism following IV injection as well as IP injection.

To further characterize the biodistribution of mRNA expression following injection of the BA-LNPs, fractional luminescence distributed amongst the liver, spleen, and all other harvested organs was plotted. Over 70% of luminescence following injection with CA-100 was in the spleen, demonstrating that CA-100 is a spleen-tropic LNP formulation that preferentially delivers to the spleen over the liver (**Figure 5D**). This analysis demonstrates that, in the context of all assessed organs and not just the liver, the spleen is still the primary target of CA-100. In other words, CA-100 demonstrates a tropism specifically for the spleen, but not other extrahepatic organs.

Given that the spleen is a secondary lymphoid organ and contains primarily lymphocytes and other immune cells, further studies were conducted to assess which cell types CA-100 was delivering mRNA to in an *in vivo* setting. Utilizing an established Cre/lox model, we observed B cells, T cells, macrophages, and dendritic cells, and we demonstrated that B cells were the only immune cells with significantly improved expression of tdTomato (the Cre/lox fluorescent reporter protein) in CA-100-treated mice as compared to S2-treated mice (**Figure S2A**) 54. B cells, notably, comprised over 50% of cells in the spleen samples, suggesting that the bulk of cells that CA-100 delivers to in the spleen are B cells (**Figure S2B**) 55. Furthermore, these results appear to recapitulate the *in vitro* screening conducted in Raji (B cells) and Jurkat (T cells) cell lines where CA-100 improved B cell delivery with no change in T cell delivery.

Interestingly, LCA-75 achieved higher liver and spleen delivery, though not statistically significant. However, it did not enhance differential delivery to either organ as the spleen:liver ratio in mice treated with LCA-75 was not significantly different than in mice treated with S2 (**Figure 5C**). LCA-75 was also the only BA-LNP candidate that significantly increased delivery following IV injection to all organs including the lungs and gastrointestinal tract (**Figure 5E**). Therefore, future research may investigate whether the LCA-75 formulation, 75% substitution of cholesterol for LCA, can serve as a more potent delivery vehicle than unmodified LNP formulations, containing only cholesterol. LCA-75 may also be further explored specifically for its potential to increase mRNA delivery to the lungs and gastrointestinal tract (stomach, small intestine, and large intestine) which may aid in the treatment of lung and small intestine disorders such as cystic fibrosis and Crohn's disease 56-58.

### Generalization of CA replacement strategy

CA-100, featuring a 100% substitution of cholesterol for CA, emerged from these studies as a spleen-tropic LNP for mRNA delivery. However, the ionizable lipid utilized in these studies, C14-4, is just one of a myriad of ionizable lipids chosen by various research groups (**Figure 5F**). Therefore, we wanted to evaluate whether the CA replacement strategy was generalizable to LNP formulations containing other ionizable lipids. Here, we chose to investigate this hypothesis with LNPs containing C12-200, a commercially available ionizable lipid utilized by several groups (**Figure 5F**) 22,28,59-62. C12-200 LNPs have demonstrated strong liver tropism *in vivo* and, therefore, have been primarily utilized for hepatic gene knockdown or delivery. Therefore, the selection of this ionizable lipid was intended to evaluate whether the CA replacement strategy achieves differential delivery to the spleen in a highly liver-tropic LNP formulation.

We formulated a C12-200 LNP containing only cholesterol (C12-Chol) and a C12-200 LNP containing only CA (C12-CA). Both formulations were screened via IV injection and organs were dissected and imaged as previously described. C12-CA achieved a 3-fold increase in fractional luminescence in the spleen compared to C12-Chol, from approximately 5% to 15% (**Figure 5G**). Though these percentages are lower than that of C14-4 LNPs, it was a statistically significant improvement and demonstrates the ability of CA replacement to shift the delivery of mRNA from the liver to the spleen.

### LNP accumulation versus functional mRNA biodistribution

The CA replacement strategy demonstrated a shift in mRNA delivery to the spleen for both C14-4 and C12-200 LNPs. However, the mechanism of action is unclear. Recently it was shown that when anionic lipids are added to LNP formulations, similar spleen tropism can be observed 30. Therefore, it is possible that the observed reduction in LNP zeta potential following the addition of bile acid may influence spleen tropism for cholic acid-substituted LNPs. Additionally, cholesterol is hypothesized to exchange with ionizable lipids from the core to the shell of the LNP during ApoE binding 63. Therefore, cholesterol plays an important role in mediating the adsorption of ApoE to the LNP surface, and subsequent uptake of ApoE-coated LNPs by low-density lipoprotein (LDL) receptors in hepatic tissue 53. Finally, we hypothesized that polymorphisms in LNPs may affect their trafficking and processing by altering LNP-protein and LNP-cell interactions. Thus, we hypothesized that a combination of negative LNP charge, protein corona, and morphology may be driving this shift.

To investigate this further, we utilized DiR-labeled LNPs (C12-Chol and C12-CA) to differentiate where LNPs were accumulating as opposed to where mRNA expression was observed. Interestingly, we found that there was no difference between C12-Chol and C12-CA in terms of accumulation of LNPs in the liver, spleen, or other organs (**Figure 5H**). Taken with the increased mRNA expression in the spleen observed with C12-CA over C12-Chol, these results suggest that C12-CA induces preferential cellular uptake within the spleen. We hypothesize that the accumulation of LNPs in the liver may be the result of the hepatic first pass, but that a combination of altered protein corona and morphology may be driving the subsequent, preferential uptake within the spleen. However, further experiments, involving an assessment of BA-LNP protein corona and the impacts of morphology of BA-LNP interactions with cell membranes, need to be conducted to determine the mechanism of action for this splenic tropism.

## Conclusion

In conclusion, we evaluated the effects of bile acid incorporation into LNPs *in vitro* and *in vivo*. These experiments revealed that BA-LNPs exhibit altered mRNA delivery patterns both *in vitro* and *in vivo*. First, several of the BA-LNPs demonstrated reduced delivery of mRNA to liver cells *in vitro* and significantly increased delivery of mRNA to other evaluated cell lines, notably T and B cells. Selected BA-LNP candidates further demonstrated biodistribution shifts *in vivo*. Specifically, CA-100, an LNP containing only CA and no cholesterol, was able to achieve significant splenic tropism following IP and IV administration compared to S2, the cholesterol-based control LNP, which, like many commercial LNP formulations, was liver tropic. Moreover, we demonstrated that the replacement of cholesterol with CA may serve as a generalizable strategy for achieving splenic tropism in other LNP formulations. With these results, it is evident that CA-100, a four-component LNP formulation, is a viable delivery vehicle for the treatment of spleen disorders with mRNA therapies. More broadly, the use of bile acids in LNP formulations has been established as a strategy for achieving shifts in the biodistribution of mRNA therapies making the targeted delivery of mRNA to extrahepatic organs more feasible.

## Supplementary Material

Supplementary figures.

## Figures and Tables

**Table 1:** LNP library characterization data.

LNP	Bile Acid Subs.	Subs. (%)	Size (nm)	PDI	EE (%)	Zeta Potential (mV)	pKa
S2	-	-	75.5 ± 1.8	0.26 ± 0.031	93.2 ± 0.4	2.6 ± 0.8	6.21
CDCA-25	CDCA	25	115.7 ± 7.3	0.237 ± 0.029	97.5 ± 0.1	-5.8 ± 2.6	6.60
CDCA-50	CDCA	50	87.4 ± 3.1	0.213 ± 0.014	87.2 ± 0.6	-1.2 ± 0.1	6.47
CDCA-75	CDCA	75	112.2 ± 5	0.213 ± 0.015	79.8 ± 0.7	-3.5 ± 0.4	6.48
CDCA-100	CDCA	100	86.4 ± 8.1	0.268 ± 0.068	48.8 ± 0.9	-8.7 ± 1.2	6.65
CA-25	CA	25	113 ± 5.7	0.227 ± 0.004	97.9 ± 0.1	-2.6 ± 2.4	6.48
CA-50	CA	50	95.5 ± 2.3	0.153 ± 0.032	96.2 ± 0.4	0.5 ± 1	6.45
CA-75	CA	75	87 ± 4.6	0.224 ± 0.015	91 ± 0.5	-4.2 ± 1.6	6.47
CA-100	CA	100	104.6 ± 1.4	0.189 ± 0.021	82.6 ± 1.5	-3.3 ± 1.5	6.39
DCA-25	DCA	25	105.9 ± 1.1	0.21 ± 0.028	93.1 ± 0.3	0.5 ± 0.5	6.54
DCA-50	DCA	50	76.7 ± 3.5	0.249 ± 0.061	90.9 ± 1.6	0.4 ± 1.4	6.39
DCA-75	DCA	75	108.5 ± 0.8	0.244 ± 0.025	72.4 ± 0.6	-3.3 ± 1.2	6.61
DCA-100	DCA	100	120.7 ± 7.3	0.236 ± 0.004	64.1 ± 1.3	-8.1 ± 1.2	6.46
LCA-25	LCA	25	110.8 ± 6.6	0.282 ± 0.031	98.4 ± 0.1	0.7 ± 0.4	6.49
LCA-50	LCA	50	84.2 ± 1.5	0.234 ± 0.016	94.5 ± 0.3	-1 ± 0.5	6.35
LCA-75	LCA	75	99.2 ± 4.5	0.234 ± 0.013	93.7 ± 0.4	-2.3 ± 0.7	6.30
LCA-100	LCA	100	118.4 ± 0.4	0.175 ± 0.034	81.9 ± 2.2	-1.8 ± 0.9	6.23

CA: cholic acid; CDCA: chenodeoxycholic acid; deoxycholic acid; EE: encapsulation efficiency; LCA: lithocholic acid; LNP: lipid nanoparticle; PDI: polydispersity index; Subs: substitution

## Abbreviations

BA-LNP: bile acid-containing lipid nanoparticle
CA: cholic acid
CDCA: chenodeoxycholic acid
DCA: deoxycholic acid
DOPE: 1,2-dioleoyl-sn-glycero-3-phosphoethanolamine
FBS: fetal bovine serum
IM: intramuscular
IP: intraperitoneal
IV: intravenous
LCA: lithocholic acid
LNP: lipid nanoparticle
mRNA: messenger ribonucleic acid
P/S: penicillin/streptomycin
PBS: phosphate-buffered saline
ApoE: apolipoprotein e
PDI: polydispersity index
TNS: 6-(p-toluidino)-2-naphthalenesulfonic acid
EM: electron microscopy
PEG: polyethylene glycol
