# Supplementary material for: Bile acid-containing lipid nanoparticles enhance extrahepatic mRNA delivery
Source: Theranostics. 2024 Jan 1;14(1):1–16. doi: 10.7150/thno.89913 (PMC10750194; doi:10.7150/thno.89913)
Supplement: Supplementary file 1 — Supplementary figures. [file thnov14p0001s1.pdf]

Title: Bile acid-containing lipid nanoparticles enhance extrahepatic mRNA delivery

Supplemental Information

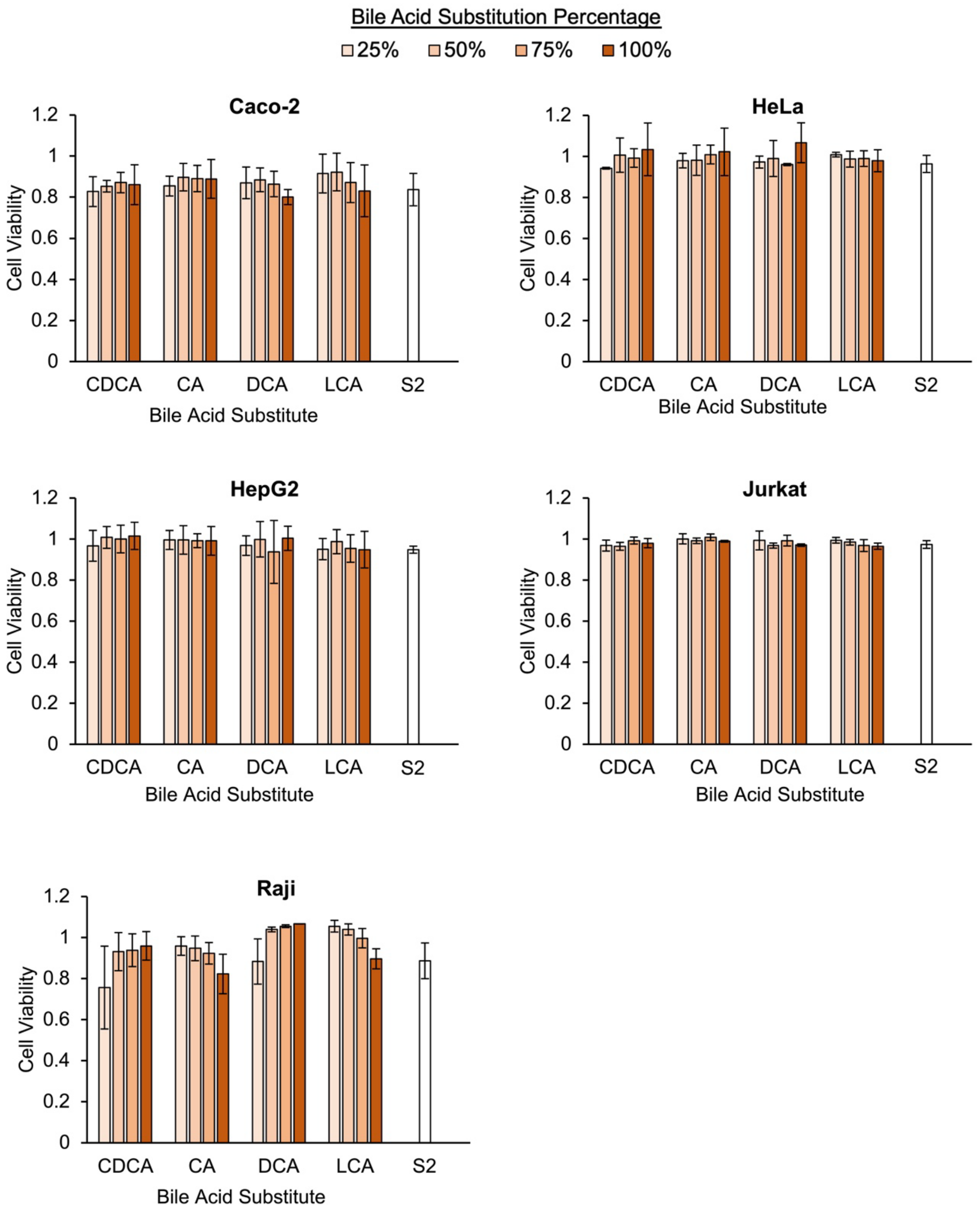

Figure S1. *In vitro* cell viability assays of the LNP library in all assessed cell lines. Luminescent expression, corresponding to cell viability, from cells treated with each BA-LNP or S2 was normalized to the baseline luminescent expression of untreated cells within each assay plate. HeLa cervical cancer cells were treated at 10 ng mRNA / 10,000 cells. HepG2 hepatocytes were treated at 10 ng mRNA / 5,000 cells. Caco-2 intestinal epithelial cells were treated at 100 ng mRNA / 25,000 cells. Jurkat T cells and Raji B cells were treated at 60 ng mRNA / 60,000 cells. Legend denotes percent

substitution of each bile acid into the S2 formulation. n = 3 biological replicates. Error bars denote standard deviation. An ANOVA was used to determine if treatment group means differed significantly. *: $p < 0.05$ in a *post hoc* Student's t-test.

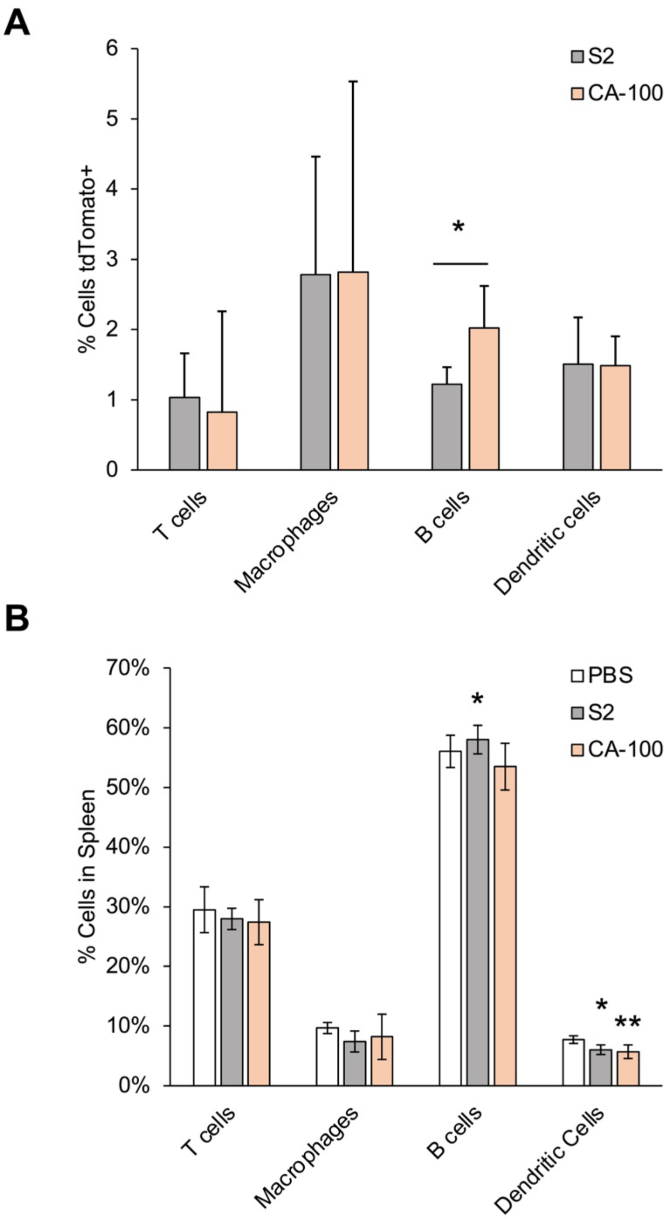

Figure S2. Cell-based flow cytometry data from Cre/lox Ai9 mouse model comparing spleens of PBS, S2, and CA-100 treated Ai9 mice. (A) Percentage of each cell type (T cells, macrophages, B cells, dendritic cells) that were tdTomato+. Treatment groups include S2 and CA-100. Reported percentages of cells expressing tdTomato+ are following subtraction of baseline values observed in PBS-treated mice. n = 4 biological replicates. Error bars denote standard deviation. An ANOVA was used to determine if treatment group means differed significantly. *: $p < 0.05$ in a *post hoc* Student's t-test. (B) Percentage of spleen samples from each treatment group (PBS, S2, and CA-100) that were gated as the various cell types (T cells, macrophages, B cells, and dendritic cells). n = 4 biological replicates. Error bars denote standard deviation. An ANOVA was used to determine if treatment group means differed significantly. *: $p < 0.05$. **: $p < 0.01$ in a *post hoc* Student's t-test between LNP treatment groups and PBS.
